# THE EFFECT OF COMBINED MOTOR AND COGNITIVE REHABILITATION ON MOTOR PERFORMANCE IN PARKINSON’S DISEASE: A SYSTEMATIC REVIEW AND META-ANALYSIS

**DOI:** 10.2340/jrm.v58.45360

**Published:** 2026-05-25

**Authors:** Luisa CACCIANTE, Alex LANDO, Emine Gulden OZCAN, Daniela D’IMPERIO, Roberto MERONI, Agnieszka GUZIK, Mariusz DRUŻBICKI, Błażej CIEŚLIK, Pawel KIPER

**Affiliations:** 1 Healthcare Innovation Technology Lab, IRCCS San Camillo Hospital, Venice, Italy; 2 Translational Rehabilitation Sciences Group, Department of Biomedical and Neuromotor Sciences (DIBINEM), Alma Mater Studiorum University of Bologna, Bologna, Italy; 3 School of Psychology, University of Padua, Padua, Italy; 4 IRCCS San Camillo Hospital, Venice, Italy; 5 Department of Health, LUNEX, Differdange; 6 Luxembourg Health & Sport Sciences Research Institute ASBL, Differdange, Luxembourg; 7 College of Medical Sciences, University of Rzeszów, Rzeszów, Poland

**Keywords:** cognition disorders, motor disorders, neurological rehabilitation, Parkinson’s disease

## Abstract

**Objective:**

To evaluate the effectiveness of sequential and simultaneous motor and cognitive treatment on motor functions, activities of daily living, and quality of life in people with Parkinson’s disease.

**Design:**

Systematic review and meta-analysis.

**Subjects/Patients:**

Patients with Parkinson’s disease.

**Methods:**

A search was conducted in PubMed, Embase, Scopus, Web of Science, and Cochrane Library. Functional mobility was assessed as the main outcome, and balance, gait, activities of daily living, and quality of life as secondary outcomes. Meta-analyses were conducted using mean difference or standardized mean difference with 95% confidence intervals and fixed or random effect models. Heterogeneity was explored, setting a cut-off value of I^2^ = 50%.

**Results:**

Sixteen studies were included, with 8 eligible for meta-analysis. Results showed that sequential combined treatment offered no clear advantage over motor rehabilitation alone for functional mobility, although a significant benefit was found for activities of daily living. Results on simultaneous combined treatment suggested improvements in quality of life, but no significant differences between treatments for functional mobility.

**Conclusion:**

Motor-cognitive approaches may enhance prefrontal cortex efficiency, supporting complex motor tasks and reducing the risk of falls. Future research should elucidate neural mechanisms and compare simultaneous and sequential strategies to develop personalized, multidisciplinary rehabilitation protocols.

## LAY ABSTRACT

Parkinson’s disease affects movement, thinking, and everyday independence. Rehabilitation programmes sometimes combine physical exercises with cognitive training, but it is unclear whether this approach is better than physical therapy alone. This study reviewed and analysed results from previous studies involving people with Parkinson’s disease. The authors compared physical rehabilitation alone with programmes that combined motor and cognitive training, focusing on walking, balance, daily activities, and quality of life. The results showed that combining motor and cognitive training did not clearly improve walking ability compared with physical therapy alone. However, people who received combined training showed a small but meaningful improvement in quality of life or daily activities, such as dressing or moving around at home. These findings suggest that motor-cognitive rehabilitation may help people with Parkinson’s disease manage everyday tasks. Further research is needed to identify effective and personalized rehabilitation approaches.

Parkinson’s disease (PD) is a progressive neurodegenerative disorder characterized by motor symptoms such as bradykinesia, rigidity, tremor, and postural instability, as well as non-motor symptoms including cognitive decline, sleep disturbances, and autonomic dysfunction (1, 2). Being the second most prevalent neurodegenerative disease among the elderly population (3), PD significantly reduces quality of life (QoL) and poses challenges for both patients and caregivers (4, 5). While pharmacological and surgical interventions, such as levodopa therapy and deep brain stimulation, remain the gold standard of treatment, their limitations – such as diminishing efficacy over time and adverse side effects – highlight the need for adjunctive strategies (6).

Physiotherapy plays a critical role in the non-pharmacological management of PD (7). It has been shown to improve mobility, balance, strength, and overall functional independence, addressing key motor impairments (7). Techniques such as balance training, resistance exercises, and gait re-education are core aspects of physiotherapy programmes for PD (7, 8). Furthermore, the use of new technologies and systems such as virtual reality (VR), exergaming, and robot-assisted training is increasing widely. According to current findings, these new approaches showed some improvements in motor symptoms and risk of falls (9, 10).

However, as PD affects more than just motor function, there is an increasing interest in therapies targeting more than one function at a time, addressing cognitive, independence, and well-being alongside physical health (11).

Complementary to motor rehabilitation, cognitive rehabilitation and cognitive training have been used to manage cognitive impairments in PD patients. Executive functions, more specifically impairments in attention, processing speed, working memory, set-shifting, and planning, characterize the cognitive profile of PD (12).

Alongside research on motor or cognitive training, studies on combined motor and cognitive treatments (MCT), whether simultaneous (i.e., the completion of motor and cognitive tasks at the same time) or sequential (i.e., performing motor and cognitive treatment sequentially) have been conducted (13). In healthy adults, combined MCT have produced the largest gains in executive functions, processing speed, and global cognition, as well as the largest improvements in motor functions (14). Nevertheless, there is insufficient data regarding how combined MCT affects motor recovery in PD. Hence, the aim of this systematic review is to assess the effect of combined MCT, both sequential and simultaneous, on motor functions in PD.

## METHODS

The research was carried out in compliance with the Preferred Reporting Items for Systematic Reviews and Meta-Analysis (PRISMA) criteria (15). It was preregistered in the International Prospective Register of Systematic Reviews (PROSPERO) under the following registration number: CRD42020191856.

### Electronic searches

A systematic electronic database search was conducted in PubMed, Embase, Scopus, Web of Science, and the Cochrane Library for papers published in English. The last search was conducted on 3 June 2025. The detailed search strategy can be found in Appendix S1.

### Study selection

Only studies published in English were included in this review. Also, studies had to meet the following inclusion criteria: (*i*) randomized controlled trials (RCT) or quasi-RCT as study design; (*ii*) all types of motor treatments combined with cognitive rehabilitation, either simultaneous or sequential, including the use of technologies; (*iii*) studies that assessed motor functions (i.e., upper and lower limb functions, balance, gait speed, functional mobility), QoL, and activities of daily living (ADL); (*iv*) studies comparing the use of combined MCT to single treatment or no intervention.

For abstract screening, 2 review authors independently screened the records obtained from the search, by using the Rayyan tool (16). The screening was performed based on title and abstract using an inclusion and exclusion criteria template. Following screening, a third reviewer was appointed to solve any disagreements between the 2 authors. After this process, full text screening was performed with the same procedure.

### Outcomes

The primary outcome of the study was the improvement of functional mobility, whereas secondary outcomes were related to the improvement of balance, gait, QoL, and ADL.

### Data extraction

An *ad hoc* synoptic table was used to extract qualitative data from the included studies. Extracted data included: participants’ demographics, study design, details of treatments provided, dosage delivered, outcome measures used in the study, findings of the study. Quantitative data were extracted as mean and standard deviations and collected in tables that were used to perform the meta-analyses. We contacted trial authors to ask for missing data (i.e., methods of randomization and/or allocation, or additional information related to results in order to carry out the meta-analyses).

### Assessment of risk of bias in included studies

The Cochrane Risk of Bias tool 2.0 (RoB2) was used to assess the methodological quality of RCTs (17). According to this tool, the following domains were assessed: bias arising from the randomization process, bias due to deviations from intended interventions, bias due to missing outcome data, bias in measurement of the outcome, bias in selection of the reported result, and overall risk of bias. In order to reach an overall risk-of-bias judgement for a specific outcome, for each domain the risk of bias was coded into 1 of the 3 following possibilities:

- Low: low risk of bias.
- High: high risk of bias.
- Some concerns: when the reporting was insufficient, and some concerns were raised.

The Joanna Briggs Institute critical appraisal tool for quasi-experimental study (18) was used to assess the methodological quality of 1 study designed as a quasi-experimental clinical trial.

After an independent assessment of the risk of bias by 2 reviewers, any inconsistencies were discussed with a third author in order to resolve them.

### Measures of treatment effect and assessment of heterogeneity

We used Review Manager (RevMan) Version 7.12.0 (https://www.cochrane.org/products-and-services/review-writing-software) to conduct statistical analysis. The treatment effects were evaluated using mean difference (MD) for homogeneous outcome measures or standardized mean difference (SMD) for analysis involving the use of different measures. The confidence interval (CI) for continuous outcomes was identified at 95%. Statistical heterogeneity was assessed with the I² statistic, establishing the cut-off value at 50%. We conducted meta-analyses based on fixed effects or random effects models with 95% CI.

## RESULTS

**Fig. 1:**
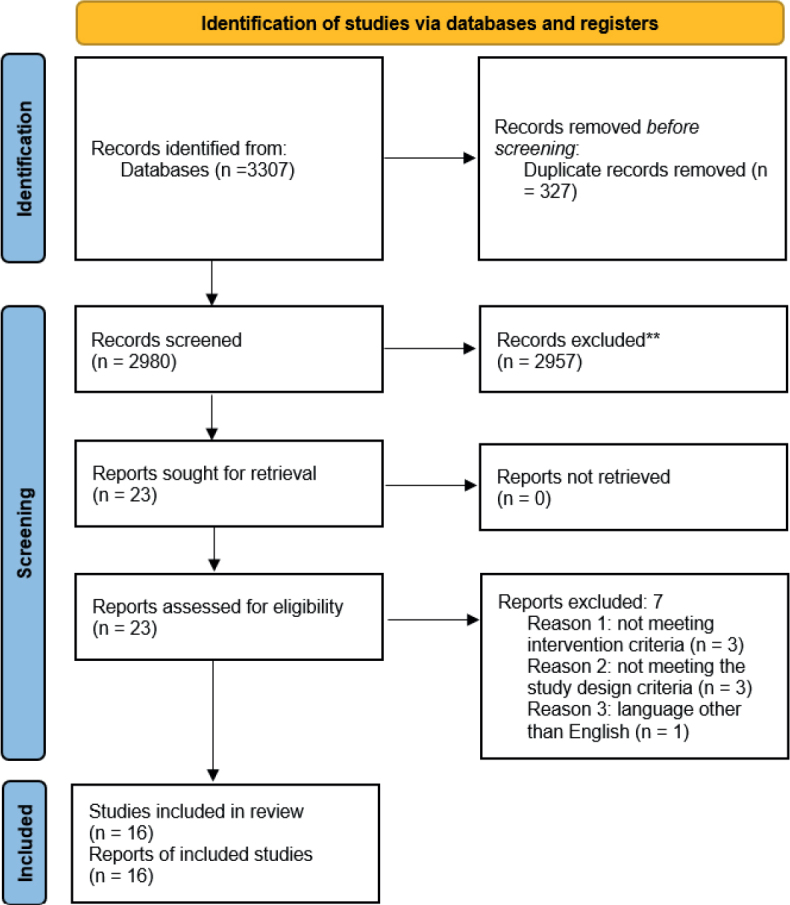
PRISMA flow diagram.

A total of 3,307 articles were retrieved via electronic searching. After removing the duplicated records, 2,980 records remained for abstract screening. After abstract screening, 23 articles were included for full text reading. Finally, 16 papers were included for qualitative evaluation, and 8 studies were quantitatively analysed. The PRISMA flow diagram is displayed in Fig. 1.

### Excluded studies

After full-text screening, 7 studies were excluded from the systematic review for the following reasons: (*i*) study was written in a language other than English (11), (*ii*) was excluded due to wrong publication type (19), 1 studies due to wrong study design (20, 21), and 3 studies were excluded due to wrong intervention type (22–24).

### Characteristics of included studies

Among the included studies, 13 were RCTs, 1 was a quasi-experimental clinical trial, and 2 were RCTs with a crossover design, all of them including patients with PD. Of the 16 studies included, 10 evaluated the effectiveness of a simultaneous motor and cognitive treatment, where motor and cognitive trainings were conducted at the same time (13), as compared with intervention groups in which cognitive and motor tasks were performed separately or no intervention was provided (9, 25–33). Only Yang and colleagues compared 2 different simultaneous dual-task training (i.e., cognitive dual-task gait training and motor dual-task gait training) with general gait training as control group (30). The remaining 6 studies assessed the effectiveness of sequential combined MCT compared with motor intervention alone (10, 34–38).

The overall number of participants included within trials was 698, with 347 patients involved in combined motor-cognitive training programmes, and 351 patients treated in control groups.

With regard to the intervention provided, 5 studies involved the use of VR and gaming systems to deliver combined treatments (9, 25, 27–29), whereas in 10 studies motor exercises (e.g., balance training, gait training) were combined with cognitive activities (e.g., calculation, attention, spatial orientation), with Del Pino and colleagues delivering sequential combined MCT through telerehabilitation (10, 26, 30–32, 34–38). In the study of Das et al., a simultaneous MCT was delivered through technological visuo-cognitive training involving the performance of motor exercises whilst wearing stroboscopic glasses (33).

The dose of therapy varies between studies, ranging from a total of 10 h of treatment overall (10 sessions over 5 weeks, 1 h per session), to a maximum of 100 h (40 sessions over 8 weeks, 2 h and 30 min per session).

A detailed description of the characteristics of included studies is presented in Table SI.

### Sequential combined motor–cognitive treatment vs motor treatment

Six included studies evaluated the effect of sequential combined motor–cognitive treatment, that is, training involving motor and cognitive tasks separately. In this context, Del Pino and colleagues evaluated the effects and the usability of a virtual coach that involved physical and cognitive telerehabilitation for patients with PD (i.e., vCare) and compared it with clinical standard care. Results showed significant improvement in the vCare group compared with the control group in cognition, QoL dimensions of mobility, self-care, daily activities, and pain/discomfort at post-treatment assessed with the EQ-5D (38). El Semary and colleagues assessed the efficacy of sequential combined MCT via Rehacom on functional outcomes, cognition, and QoL in PD, as compared with physical therapy alone. Significant improvements in functional outcomes and QoL were shown in the experimental group (EG) (10). Mariano Barboza et al. aimed to verify the effectiveness of physiotherapy associated with cognitive training as compared with physiotherapy alone to improve cognition and QoL in individuals with PD. In this case, the intragroup analysis revealed that both groups presented improved performance in memory and visuospatial function, together with a better QoL after execution of the training, but without statistically significant intergroup differences (34). Monticone et al. proposed an intervention protocol involving motor and cognitive training and ergonomic education, and evaluated its effects on motor impairment, ADL, and QoL, as compared with general physiotherapy. They found between-group significant differences in favour of the EG for motor outcome, demonstrating superiority of a multidisciplinary rehabilitative programme compared with general physiotherapy (35). Terra and colleagues proposed to evaluate the efficacy of physiotherapy plus cognitive training compared with physiotherapy alone on balance in PD, and they did not find differences between these 2 treatments, even if both interventions improved patients’ balance and signs and symptoms of PD (36). Finally, Varalta et al. investigated the effects of sequential combined MCT on cognitive, motor, and psychological aspects in PD patients, as compared with 3 months of physiotherapy. They found significant differences in functional mobility between the 2 groups in favour of the EG, concluding that combined treatment may have a greater benefit than physiotherapy alone in patients with PD (37).

### Simultaneous motor–cognitive treatment versus motor treatment

Simultaneous motor–cognitive training is defined as training where both motor and cognitive training are performed at the same time (13). We included 10 studies focused on assessing the effectiveness of simultaneous MCT as compared with single-task training.

Among the included studies, Alves et al. compared the use of 2 different commercial gaming devices (i.e., Nintendo Wii™ and Xbox Kinect™) for motor and cognitive training, with a third control group not receiving intervention. Results from their study showed that only participants engaged with the Nintendo Wii™ significantly improved their performance on single- and dual-task gait tests, and had decreased anxiety levels, and improved memory, attention, and reversibility (25). Evidence of superior improvements for dual-task training compared with the single-task training for static postural control comes from the study of Fernandes et al. (26), who analysed the efficacy of simultaneous MCT compared with single-task training on balance and executive functions in patients with PD. Chua et al. (9) assessed the feasibility and safety of the use of neurorehabilitation technology (i.e., SMARTfit^®^ Trainer system) in implementing a gamified dual-task training paradigm, together with evaluation of the efficacy of this gamified dual-task training as compared with single-task training. Gamified dual-task training turned out to be safe and feasible, showing improvements in more outcome measures than single-task training related to motor functions, cognitive functions, and perceived disability (9). Lau et al. (27) introduced the use of immersive treadmill training for providing combined MCT and determined its feasibility and preliminary efficacy on gait and cognition. The authors concluded that immersive gaming technology used to engage specific areas of cognition related to gait is feasible and can improve walking velocity, in addition to non-significant but consistent improvements in other gait parameters and cognitive performance. In a similar way, Maidan and colleagues investigated whether treadmill training with VR or treadmill training alone differentially affect prefrontal activation and if this might explain differences in fall rates in participants with PD. Interestingly, they found that treadmill training with VR reduced prefrontal activation to a greater extent than treadmill training alone, and a lower number of falls after training was associated with reduced prefrontal activation in both arms, highlighting beneficial effects on fall rates of treadmill training with VR targeting both motor and cognitive functions (28). Conversely, Pompeu et al. (29) did not find significant advantages associated with the combined MCT on ADL, when using Wii-based technology as compared with balance exercise therapy. Das and colleagues determined the feasibility and preliminary efficacy of a home-based, technological visuo-cognitive training intervention and exercise with stroboscopic glasses compared with non-technological, standard care in patients with PD. The authors found improvements in participants’ performance in both groups for a variety of clinical, cognitive, and motor outcomes, together with good feasibility results, concluding that home-based technological visuo-cognitive training is feasible and a viable solution for addressing cognitive and motor dysfunctions in PD (33). Lin et al. aimed to compare the effects of Dual cognitive-walking Treadmill Training (DTT) vs single treadmill training (STT) on cognitive and walking performance under both single- and dual-task conditions, as well as on falling, QoL, and patients’ feelings. DTT consisted of a treadmill training with cognitive tasks displayed on a big screen in front of the treadmill. Both the DTT and STT groups showed increased comfortable walking speed and step length, but only the DTT group demonstrated significant improvements in cognitive composite score, as well as UPDRS-III, FES, and PDQ-39 compared with pre-training (32). Similarly, Wong and colleagues wanted to investigate the effects on obstacle walking and brain activities in PD of 2 novel dual-task training types, that is, motor–cognitive training and complex walking training, both challenging motor and cognitive functions. They compared the 2 training groups with a third group receiving no additional training, showing that the cognitive–motor training group improved obstacle walking performance with increased supplementary motor area activities in people with PD. Conversely, the complex walking training did not lead to such beneficial effects as the cognitive–motor training (31). Finally, Yang et al. wanted to investigate the effects of cognitive and motor dual-task gait training on dual-task gait performance in PD. Participants were assigned to 3 different groups, consisting of motor dual-task training, cognitive dual-task training, and a control group. Participants in the motor dual-task training group were instructed to perform motor tasks during different walking conditions on a level surface, whereas participants in the cognitive dual-task training group had to execute cognitive tasks during diverse walking conditions. Participants in the control group performed general gait training on a level surface. Findings from their study showed that cognitive dual-task training significantly decreased double support time during cognitive dual-task walking, and that motor dual-task training significantly reduced gait variability during motor dual task walking as compared with the control group, suggesting that different training strategies can be adopted for possibly different training effects in people with PD (30).

### Risk of bias in the included studies

Fifteen studies were evaluated for methodological quality with the RoB2 tool for RCTs with the following results:

- Bias arising from the randomization process: 10 studies were assessed with a low risk of bias, as the authors described a correct randomization process and no differences between intervention groups related to this process were detected (26, 27, 29–32, 34–37). One study (38) was judged with a high risk of bias, as participants were distributed nonrandomly. Four studies were judged with some concerns of risk of bias, as not enough information was provided (9, 10, 28, 33).
- Bias due to deviations from the intended interventions: Thirteen studies had a low risk of bias in this domain (9, 27–38). Moreover, 1 study (26) had a high risk of bias because an appropriate analysis was not used to estimate the effect of assignment to intervention and a rate of 30% in the single-task group and 20% in the dual-task group dropped out. This could have altered the data analysis. Finally, 1 study (10) did not provide information, resulting in some concerns of risk of bias.
- Bias due to missing outcome data: all studies except 1 had a low risk of bias in this domain. Only the study conducted by Terra and colleagues was judged to have a high risk of bias because almost 26% of participants dropped out in post-intervention assessment, and no information on analyses conducted to correct for bias was provided (36).
- Bias in measurement of the outcome: 10 studies had a low risk of bias in this domain (9, 26–31, 34, 35, 37). Studies by El Semary et al. and Terra et al. had a high risk of bias because, in 1 case, no information regarding blinding of outcomes assessment was provided (10), and in the other case the outcome assessor was not blinded (36). Three studies did not provide information on outcomes assessment and were judged to raise some concerns (32, 33, 38).
- Bias in selection of the reported result: All studies but 1 were judged to have some concerns regarding the presence of risk of bias, because no information on study protocol or data analysis plan was provided. Only Chua et al. provided a protocol and a data analysis plan in accordance with the reported results (9).
- Overall bias: None of the studies was judged to have a low risk of bias. Four studies presented a high risk of bias due to problems in randomization (38), deviations from intended intervention (26), missing outcome data (36), and in measurement of the outcome (10, 36). The remaining 11 studies were judged to raise some concerns regarding the presence of bias, mostly due to no information provided for the selection of the reported results.

Fig. S1 shows the risk of bias in the included studies.

One study (25) was assessed with the JBI tool for quasi-experimental studies and no risk of bias was detected. Table SII shows the evaluation of the methodological quality of the study according to the tool.

## EFFECTS OF INTERVENTION

### Comparison 1. Sequential combined motor–cognitive treatment vs motor treatment

**Fig. 2:**
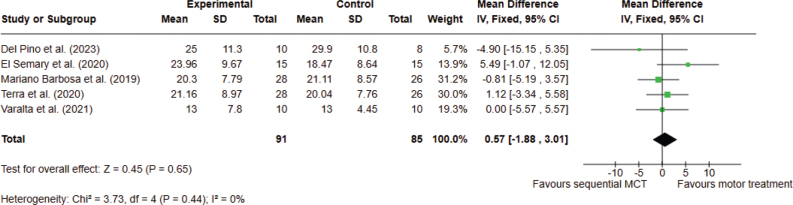
Comparison 1. Sequential combined motor–cognitive treatment vs motor treatment. Outcome 1.1 Functional mobility. A lower score means an improvement in the outcome.

*Analysis 1.1. Effect on functional mobility – post intervention.* Functional mobility was operationalized using the UPDRS-III motor examination. Although this scale primarily assesses motor impairment, it includes key components such as bradykinesia, rigidity, and postural stability, which are major determinants of mobility performance in PD, and its scores have been shown to correlate with objective mobility measures such as gait and Timed Up and Go performance (39). A total of 5 studies, with 176 participants overall, were analysed. The meta-analysis did not provide significant evidence that sequential combined motor–cognitive treatment is superior to conventional motor treatment for functional mobility assessed with the UPDRS-III. The analyses were performed using MD with a fixed effects model. No significant differences were found between the 2 types of treatments (MD 0.57; 95% CI –1.88 to 3.01, I^2^ = 0%) (Fig. 2).

### Comparison 1. Sequential combined motor-cognitive treatment vs motor treatment

**Fig. 3:**
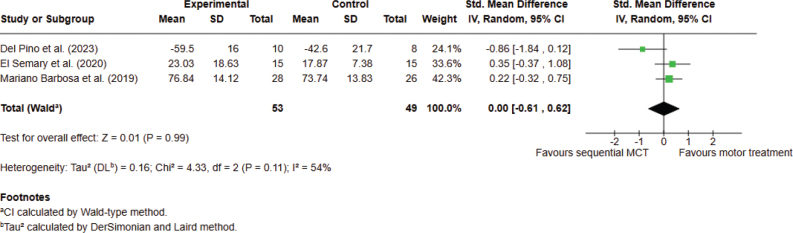
Comparison 1. Sequential combined motor–cognitive treatment vs motor treatment. Outcome 1.2. Quality of life. A lower score means an improvement in the outcome.

*Analysis 1.2. Effect on quality of life – post intervention.* A total of 3 studies, with 102 participants overall, were analysed. The meta-analysis did not provide significant evidence that sequential combined motor-cognitive treatment is superior to conventional motor treatment for improving QoL. The analyses were performed using SMD with a random effects model. No difference was found between the 2 types of treatments (SMD 0.00; 95% CI –0.61 to 0.62, I^2^ = 54%) (Fig. 3).

**Fig. 4:**
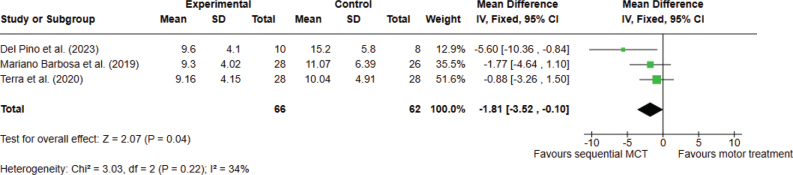
Comparison 1. Sequential combined motor–cognitive treatment vs motor treatment. Outcome 1.3. Activities of daily living. A lower score means an improvement in the outcome.

*Analysis 1.3. Effect on activities of daily living – post intervention.* Three studies, with 128 participants overall, were analysed. The meta-analysis showed significant evidence that sequential combined MCT can improve ADL as compared with conventional motor treatment, when assessed with the UPDRS-II. The analyses were performed using MD with fixed effects model. A significant difference was found between the 2 types of treatments in favour of combined MCT (MD –1.81; 95% CI –3.52 to –0.10, I^2^ = 34%) (Fig. 4).

### Comparison 2. Simultaneous combined motor–cognitive treatment vs motor treatment

**Fig. 5:**
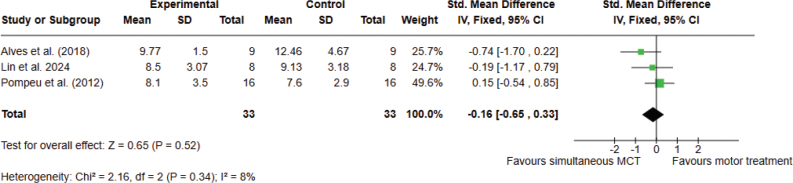
Comparison 2. Simultaneous combined motor–cognitive treatment vs motor treatment. Outcome 2.1 Functional mobility. A lower score means an improvement in the outcome.

*Analysis 2.1. Effect on functional mobility – post intervention.* A total of 3 studies, with 66 participants overall, were analysed. The meta-analysis did not provide significant evidence that simultaneous combined MCT is superior to conventional motor treatment for functional mobility. The analyses were performed using SMD with a fixed effects model. No significant differences were found between the 2 types of treatments (SMD –0.16; 95% CI –0.65 to 0.33, I^2^ = 8%) (Fig. 5).

**Fig. 6:**
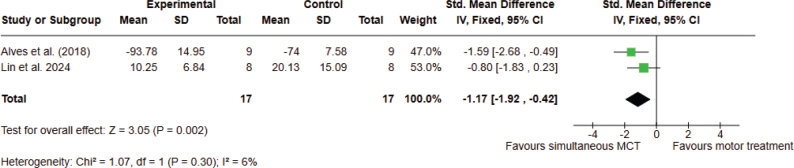
Comparison 2. Simultaneous combined motor–cognitive treatment vs motor treatment. Outcome 2.2 Quality of life. A lower score means an improvement in the outcome.

*Analysis 2.2. Effect on quality of life – post intervention.* Two studies, with 34 participants overall, were analysed. The meta-analysis did provide significant evidence that simultaneous combined MCT is superior to conventional motor treatment for improving QoL in patients with PD. The analyses were performed using SMD with a fixed effects model. A significant difference was found between the 2 types of treatments in favour of combined MCT (SMD –1.17; 95% CI –1.92 to –0.42, I^2^ = 6%) (Fig. 6).

## DISCUSSION

This systematic review evaluated the effect of combined MCT, either sequential or simultaneous, on motor functions, ADL, and health-related QoL in PD patients. Nowadays, the theoretical premises for proposing the use of combined MCT in patients with PD appear to be of clinical interest. There is evidence supporting the hypothesis that motor recovery engages large-scale brain networks that involve cognitive abilities (40), which can affect motor relearning, playing a larger role in motor recovery than previously understood (41). For instance, attention deficits, in particular visuospatial orientation, may be crucial to motor recovery, enhancing patients’ involvement during rehabilitation sessions and the ultimate result (40). Based on these considerations, it seems clear that motor rehabilitation therapies may benefit from the addition of cognitive training (37), opening up the possibility to deeply study the effects of combined treatments for motor rehabilitation.

Therefore, analysing the effects of sequential combined MCT, meta-analyses did not demonstrate clear superiority over motor training alone for functional mobility or QoL, whereas a modest but significant benefit emerged for ADL. These findings suggest that, within the relatively low treatment doses applied, adding cognitive sessions sequentially may support the transfer of motor abilities into everyday performance, improving the independence of patients with PD. It should be considered, however, that the interventions included in the pooled analyses differed substantially in terms of content, delivery modality (e.g., conventional physiotherapy, virtual reality-based training, or telerehabilitation), and treatment dose. Such heterogeneity, and the methodological limitations of the included studies, may have weakened the potential effects of specific intervention types and therefore contributed to the absence of significant pooled differences between combined and motor-only treatments.

One important aspect to take into account concerns the brain repair mechanisms activated during rehabilitation. Experimental and clinical studies of exercise and rehabilitation indicate a dynamic interplay between degenerative and regenerative processes, with activity‑dependent mechanisms modulating dopaminergic and glutamatergic neurotransmission and helping to normalize cortically driven hyperexcitability (42, 43). Moreover, previous studies have shown exercise‑-related neuroplastic changes in dopaminergic signalling, grey matter structure, circulating neurotrophic factors, cerebrovascular risk profiles, tissue oxygenation, and large‑scale brain connectivity, with effects in some cases comparable to pharmacological treatment (44, 45). Therefore, exercise-induced brain plasticity is likely to represent the neural basis of rehabilitation for PD (34, 46). Taken together, these findings highlight the possibility of combined MCT to target and activate these repair mechanisms, offering a unique opportunity to integrate multidisciplinary care while obtaining beneficial effects on cognitive and motor functions for patients with PD (34).

In relation to quantitative synthesis of simultaneous motor–cognitive protocols, results of our analyses showed a greater improvement in QoL after dual-task training as compared with single-task, although this result should be considered preliminary, as it was derived from only 2 small studies and therefore should be viewed primarily as hypothesis-generating rather than conclusive evidence. Importantly, these findings must also be interpreted in light of the methodological limitations of the available trials. As most studies were judged to have “some concerns” regarding risk of bias, particularly in the selection of reported results, the statistical significance observed for QoL outcomes should not be considered definitive evidence of efficacy. However, Lin et al. suggested that dual cognitive–walking training effects could improve the ability to coordinate different interfering tasks, which is a crucial ability for everyday life, reflecting therefore an improvement in perceived health-related QoL (32). Speaking about motor functions, meta-analysis did not show significant differences between dual- and single-task training for functional mobility, but several trials consistently reported improvements in dual‑task gait, postural control and, in some cases, fall outcomes (9, 26, 30, 31). An interesting explanation for the benefits of combined MCT for walking ability comes from the study of Maidan and colleagues, who evaluated the effects of the combined treatment using virtual reality-based paradigms on prefrontal activation during walking (28). Authors found that MCT reduced prefrontal activation to a greater extent than a usual walking condition, and that, in turn, reduced prefrontal activation was related to a lower number of falls. Taken together, these findings could explain the benefits of dual-task training, suggesting that MCT may lead to a more efficient recruitment of the prefrontal cortex during complex walking conditions (28). These data support the hypothesis that simultaneous dual-task practice targets neural mechanisms that are not engaged when motor and cognitive training are delivered sequentially.

Another key element that needs to be highlighted refers to the dose of treatment provided in the studies analysed. The question of the amount of treatment to be delivered in order to observe benefits from various rehabilitative approaches is a very thorny one, and clashes with the possibilities of health services to be able to deliver high-dose treatments and with the expectations of clinicians from rehabilitative treatments for different kinds of diseases (e.g., stroke or neurodegenerative diseases). This is also reflected in the studies included in this review, in which the doses of treatment delivered vary from 10 h to a maximum of 100 h, without observing a consistency between the dose of treatment delivered and clinical changes in the outcomes assessed. Researchers should therefore focus on finding the optimal dose of treatment to be delivered to patients with neurodegenerative disorders, as they differ clinically from, for instance, patients with stroke, for whom some evidence is emerging on the optimal dose of treatments to be delivered to make significant changes (47, 48).

Taken together with the neutral findings on sequential protocols, these patterns indicate that the temporal structure of motor–cognitive dosing (simultaneous vs sequential) may be as important as total training time and should be considered a key design variable in future trials. Indeed, previous studies showed that dual-task training can promote automatic movement and can reduce the interference of a second task on the primary motor task one is performing (30, 49). That is, if attention is devoted to a cognitive task during motor training, it may prohibit the use of compensatory cognitive strategies and may lead patients with PD to be more reliant on the striatal motor pathway during movement execution, which in turn may promote the restitution of the impaired striatal motor pathway (50, 51). Research should therefore focus on evaluating the difference between sequential and simultaneous dual-task training, in order to move towards more personalized and multidisciplinary rehabilitation strategies, exploiting the potential of multi-domain rehabilitation strategies.

### Limitations

A first limitation of this review is related to the low number of articles included for the meta-analyses on the effects of the simultaneous combined treatment, which reduced the reliability and interpretability of the results. Together with this aspect, it needs to be highlighted that the low number of participants included in the meta-analyses makes it difficult to draw firm conclusions on this topic. Beyond small sample sizes and limited statistical power, another limitation lies in the presence of heterogeneity due to the interventions themselves, which ranged from low‑dose, centre‑based cognitive components to intensive, technology‑driven dual‑task protocols.

Taken together, these limitations suggest on the one hand that the results of the performed meta-analyses should be considered with caution, whereas on the other hand they underline the current gaps in the literature in this field, providing insights for future research on combined MCT.

In conclusion, combined MCT did not show clear superiority over conventional motor rehabilitation for functional mobility in people with PD, although sequential protocols yielded a small but significant advantage for ADL. These findings suggest that adding cognitive components to physiotherapy may help patients translate motor abilities into everyday function. Evidence from simultaneous dual‑task interventions indicates potential benefits for health‑related QoL, dual‑task gait performance, and falls, possibly via more efficient recruitment of prefrontal networks during complex walking. Taken together, current data support the clinical rationale for integrating cognitive and motor elements in PD rehabilitation, but also highlight that the temporal structure, content, and dose of combined interventions remain insufficiently defined. Nevertheless, given the limited number of trials and the relatively small sample sizes included in the meta-analyses, these findings should be interpreted as preliminary signals that warrant confirmation in larger, well-designed randomized trials. Future trials should directly compare simultaneous vs sequential formats and clarify how MCT can be optimized to improve mobility, independence, and QoL in people with PD.

Understanding the interaction between cognitive and motor variables and how this interaction could be harnessed by multi-domain intervention strategies is imperative for optimizing rehabilitation treatments. By exploiting the insights gained from combining motor and cognitive interventions, there is potential for enhancing the effectiveness of rehabilitation approaches for PD patients and improving their overall QoL.

## Supplementary Material






