# Supplementary material for: THE EFFECT OF COMBINED MOTOR AND COGNITIVE REHABILITATION ON MOTOR PERFORMANCE IN PARKINSON’S DISEASE: A SYSTEMATIC REVIEW AND META-ANALYSIS
Source: J Rehabil Med. 2026 May 25;58:45360. doi: 10.2340/jrm.v58.45360 (PMC13213410; doi:10.2340/jrm.v58.45360)
Supplement: Supplementary file 1 [file JRM-58-45360-s1.pdf]

Supplementary material has been published as submitted. It has not been copyedited, or typeset by Journal of Rehabilitation Medicine
APPENDIX S1 – SEARCH STRATEGY

PubMed
1. ("Parkinson Disease"[Mesh] OR "Parkinsonian Disorders"[Mesh]) OR (Parkinson*)
2. ((("Cognitive exercise*" OR "Cognitive training*" OR "Cognitive rehabilitation" OR "Cognitive Therap*") OR ("Neuropsychological treatment*")) OR ("Neuropsychological training*")) OR ("Cognitive treatment*")
3. (((((((((((((("motor treatment*") OR (Physical and Rehabilitation Medicine [Mesh])) OR ("upper extremity" [Mesh])) OR ("lower extremity" [Mesh])) OR (Gait [Mesh])) OR "gait training" OR ("postural balance"[MeSH Terms])) OR (physiotherapy)) OR (rehabilitation)) OR ("physical therapy")) OR ("motor imagery")) OR ("action observation")) OR ("graded motor imagery")) OR ("virtual reality")) OR (robot*)) OR ("gait training")) OR ("dual task")) OR (combine*)

Embase
1. ('parkinson disease'/exp OR 'parkinson disease' OR 'parkinsonian disorders'/exp OR 'parkinsonian disorders' OR parkinson*) AND [embase]/lim
2. ('cognitive exercise*' OR 'cognitive training*' OR 'cognitive rehabilitation'/exp OR 'cognitive rehabilitation' OR 'cognitive therap*' OR 'neuropsychological treatment*' OR 'neuropsychological training*' OR 'cognitive treatment*') AND [embase]/lim
3. ('motor treatment*' OR 'physical and rehabilitation medicine'/exp OR 'physical and rehabilitation medicine' OR 'upper extremity'/exp OR 'upper extremity' OR 'lower extremity'/exp OR 'lower extremity' OR 'gait'/exp OR gait OR 'postural balance'/exp OR 'postural balance' OR 'physiotherapy'/exp OR physiotherapy OR 'rehabilitation'/exp OR rehabilitation OR 'physical therapy'/exp OR 'physical therapy' OR 'motor imagery'/exp OR 'motor imagery' OR 'action observation'/exp OR 'action observation' OR 'graded motor imagery'/exp OR 'graded motor imagery' OR 'virtual reality'/exp OR 'virtual reality' OR robot* OR 'gait training'/exp OR 'gait training' OR 'dual task'/exp OR 'dual task' OR combine*) AND [embase]/lim
4. #1 AND #2 AND #3

Scopus
"parkinson disease" OR "parkinsonian disorders" OR parkinson*
AND
"cognitive exercise*" OR "cognitive training*" OR "cognitive rehabilitation" OR "cognitive therap*" OR "neuropsychological treatment*" OR "neuropsychological training*" OR "cognitive treatment*"
AND
"motor treatment*" OR physical AND rehabilitation AND medicine OR "upper extremity" OR "lower extremity" OR gait OR "gait training" OR "postural balance" OR physiotherapy OR rehabilitation OR "physical therapy" OR "motor imagery" OR "action observation" OR "graded motor imagery" OR "virtual reality" OR robot* OR "gait training" OR "dual task" OR combine*

Web of Science
WC=(Rehabilitation)
TS=("parkinson disease" OR "parkinsonian disorders" OR parkinson*)
TS=("cognitive exercise*" OR "cognitive training*" OR "cognitive rehabilitation" OR "cognitive therap*" OR "neuropsychological treatment*" OR "neuropsychological training*" OR "cognitive treatment*")
TS=("motor treatment*" OR physical AND rehabilitation AND medicine OR "upper extremity" OR "lower extremity" OR gait OR "gait training" OR "postural balance" OR physiotherapy OR rehabilitation OR "physical therapy" OR "motor imagery" OR "action observation" OR "graded motor imagery" OR "virtual reality" OR robot* OR "gait training" OR "dual task" OR combine*)
#1 AND #2 AND #3 AND #4

Cochrane Library
#1 MeSH descriptor: [Parkinson Disease] explode all trees
#2 MeSH descriptor: [Parkinsonian Disorders] explode all trees
#3 (Parkinson*):ti,ab,kw
#4 (#1 OR #2 OR #3)
#5 ("cognitive exercise*" OR "cognitive training*" OR "cognitive rehabilitation" OR "cognitive therap*" OR "neuropsychological treatment*" OR "neuropsychological training*" OR "cognitive treatment*"):ti,ab,kw
#6 MeSH descriptor: [Physical and Rehabilitation Medicine] explode all trees
#7 MeSH descriptor: [Upper Extremity] explode all trees
#8 MeSH descriptor: [Lower Extremity] explode all trees
#9 MeSH descriptor: [Gait] explode all trees
#10 MeSH descriptor: [Postural Balance] explode all trees
#11 ("motor treatment*" OR "gait training" OR physiotherapy OR rehabilitation OR "physical therapy" OR "motor imagery" OR "action observation" OR "graded motor imagery" OR "virtual reality" OR robot* OR "gait training" OR "dual task" OR combine*):ti,ab,kw
#12 (#6 OR #7 OR #8 OR #9 OR #10 OR #11)
#13 (#4 AND #5 AND #12)
