# Supplementary material for: THE EFFECT OF COMBINED MOTOR AND COGNITIVE REHABILITATION ON MOTOR PERFORMANCE IN PARKINSON’S DISEASE: A SYSTEMATIC REVIEW AND META-ANALYSIS
Source: J Rehabil Med. 2026 May 25;58:45360. doi: 10.2340/jrm.v58.45360 (PMC13213410; doi:10.2340/jrm.v58.45360)
Supplement: Supplementary file 2 [file JRM-58-45360-s2.pdf]

Supplementary material has been published as submitted. It has not been copyedited, or typeset by Journal of Rehabilitation Medicine

Figure S1. Risk of bias assessment in randomised controlled trials

Study ID	D1	D2	D3	D4	D5	Overall
Chua et al. (2021)	!	+	+	+	+	!
El Semary et al. (2020)	!	!	+	-	!	-
Fernandes et al. (2018)	+	-	+	+	!	-
Lau et al. (2022)	+	+	+	+	!	!
Maidan et al. (2018)	!	+	+	+	!	!
Mariano Barboza et al. (2019)	+	+	+	+	!	!
Monticone et al. (2015)	+	+	+	+	!	!
Pompeu et al. (2012)	+	+	+	+	!	!
Terra et al. (2020)	+	+	-	-	!	-
Varalta et al. (2021)	+	+	+	+	!	!
Yang et al. (2019)	+	+	+	+	!	!
Das et al. (2024)	!	+	+	!	!	!
Del Pino et al. (2023)	-	+	+	!	!	-
Lin et al. (2024)	+	+	+	!	!	!
Wong et al. (2024)	+	+	+	+	!	!

+	Low risk
!	Some concerns
-	High risk

D1	Randomisation process
D2	Deviations from the intended interventions
D3	Missing outcome data
D4	Measurement of the outcome
D5	Selection of the reported result
