# Supplementary material for: THE EFFECT OF COMBINED MOTOR AND COGNITIVE REHABILITATION ON MOTOR PERFORMANCE IN PARKINSON’S DISEASE: A SYSTEMATIC REVIEW AND META-ANALYSIS
Source: J Rehabil Med. 2026 May 25;58:45360. doi: 10.2340/jrm.v58.45360 (PMC13213410; doi:10.2340/jrm.v58.45360)
Supplement: Supplementary file 3 [file JRM-58-45360-s3.pdf]

Supplementary material has been published as submitted. It has not been copyedited, or typeset by Journal of Rehabilitation Medicine

Table SI. Characteristics of included studies

Authors (year)	Study design	Groups characteristics (n)	Interventions	Total number of sessions; duration; session length	Outcomes	Study conclusions
**Sequential combined motor-cognitive treatment vs. motor treatment**						
Del Pino et al., 2023	RCT	EG = 10<br>CG = 10	EG: vCare TR with cognitive and motor activities targeting attention, executive functions, mobility, strength, coordination, dexterity, speed, motor control, postural control, balance, endurance, rhythm<br>CG: clinical standard at the clinic	4 times/week for 4 months (2 days for motor and 2 days for cognitive sessions). Each session approximately of 30min	EQ5D-5L, MoCA, UPDRS, H&Y, ADL	Significant improvement in QoL, cognition, motor symptoms and daily life activities for the vCare group compared to the CG. The vCare system seems to be an optimal and cost-effective tool for TR in PD.
El Semary et al. (2020)	RCT	EG = 15<br>CG = 15	EG: Cognitive rehabilitation via Rehacom and selected physical therapy program including trunk rotation & extension, weight shifting, and reciprocal motion exercises<br>CG: The same selected physical therapy program with three intermittent rest	36 sessions; 12 weeks; 90 min	VM Learning, VM Recall, VIM Learning, VIM Recall, EFs Stroop; ToM, UPDRS, PDQ-39, WHO-DAS-II	Significant improvement of functional outcomes and life quality have been showed in the EG group. Neuropsychological battery, UPDRS, PDQ-39, WHO-DAS-II are the scores that have been improved post treatment in EG group compared to CG.
Mariano Barboza et al. (2019)	RCT	EG = 29<br>CG = 29	EG: **Cognitive-motor group** = After CG Intervention, cognitive stimulation activites memory activities like calculation, concentration, and spatial orientation<br>CG: **Motor group** = Intervention focused on balance training, sensory integration, agility and motor coordination, exploration of limits of stability, anticipatory and reactive postural adjustments, functional independence, and gait improvement	EG: 32 sessions; 16 weeks; 60 min motor + 30 min cognitive CG: 32 sessions; 4 months; 60 min	UPDRS, H&Y, GDS, SVF, RAVLT, TMT, CDT, PDQL	The intragroup analysis revealed that both groups presented improved cognition (memory and visuospatial function domains) and quality of life after execution of the protocols, but without statistically significant intergroup differences.
Monticone et al. (2015)	RCT	EG = 35<br>CG = 35	EG: Motor training, cognitive training, and ergonomic education<br>CG: General physiotherapy	EG: 40 sessions; 8 weeks; 90 min physical training, 30 min cognitive trainin, 30 min ergonomic education<br>CG: 40 sessions; 8 weeks; 90 min	MDS-UPDRS-Part III, BBS, FIM, PDQ-39	The findings suggest that multidisciplinary rehabilitative care is useful in changing the course of motor impairment, balance, activities of daily living, and QoL. The effects lasted for at least 1 y after the intervention.
Terra et al. (2020)	RCT	EG = 28<br>CG = 26	EG: Physiotherapy plus Cognitive Training Group (PCG)	32 sessions; 16 weeks; PCG 60	BESTest, ADL, UPDRS-III	There was no difference between the proposed treatments (PCG and PG). However, both

			CG: Physiotherapy group (PG)	min+ 30 min cognitive training; PG 60 min		interventions improved the individuals' balance and signs and symptoms of PD, when considering the time effect.
Varalta et al. (2021)	RCT- Cross over design	EG = 10<br>CG = 10	EG: Physiotherapy and cognitive treatment separately (PCT)<br>CG: Physiotherapy treatment (PT)<br>(switch after 1 month wash out period between the two groups)	EG: 12 sessions of PT + 12 sessions of CT; 12 weeks; 50 min PT, 50 min CT<br>CG: 24 sessions of PT; 12 weeks; 50 min	MoCA, UPDRS III, FAB-it, TMT, FAS, DSF, DSB, RAVL, BDI, STAI, BBS, 2WMT, TUG	The findings support the hypothesis that consecutive physiotherapy plus cognitive rehabilitation may have a greater benefit than physiotherapy alone in patients with PD.
**Simultaneous motor-cognitive treatment vs. motor treatment**						
Alves et al. (2018)	Quasi-experimental clinical trial	EG1 = 9<br>EG2 = 9<br>CG = 9	EG1: Training with Nintendo WiiTM (both motor&cognitive)<br>EG2: Training with Xbox KinectTM<br>CG: No intervention (motor cognitive)	10 sessions; 5 weeks; 45-60 min	TUG, 10MWT, 30-Seconds Walk Test, DSB, DSF,<br>VFT<br>WAIS-III<br>BAI<br>WHOQOL_OLD	The results of the study show that only EG1 showed significant performance improvement on single and dual-task gait tests, and improvement on memory, attention, reversibility, and decreased anxiety levels.
Chua et al. (2021)	Randomized Cross-over study design	G1 = 5<br>G2 = 4	G1: Gamified Physical-Cognitive DTT by SMARTfit - Physical STT<br>G2: Physical STT- Gamified Physical-Cognitive DTT by SMARTfit	24 sessions; over 8.8 weeks; average washout period = 11.5 weeks	MDS-UPDRS II,<br>MDS-UPDRS III,<br>M-PPT,<br>PD-CRS,<br>TMT	Significant improvements were achieved in more outcome measures after gamified DTT than they were after STT.
Das et al., 2024	RCT	EG = 20<br>CG = 20	EG: TVT – Technological visuo-cognitive training. Visuo-cognitive training drills performed on a mobile tablet device and exercises whilst wearing stroboscopic glasses.<br>CG: SC - standard, non-technological care. Paper-based visuo-cognitive training tasks and seated game activities plus exercises under normal visual conditions.	1 hour/session, twice/week for 4 weeks	UPDRS, PDQ-39, FES-I, FSS, MiniBesTest, TUG; TMT, SRT, FDS	Participants in both groups improved in a variety of clinical, cognitive, and physical performance outcomes. Home-based TVT is feasible and could provide an approach addressing cognitive and motor dysfunction in patients with PD.
Pompeu et al. (2012)	RCT	EG = 16<br>CG = 16	EG: Wii-based motor and cognitive training<br>CG: Balance exercise therapy	14 sessions; 7 weeks; 60 min each	UPDRS-II, BBS, UPST (eyes open and eyes closed), MoCA	Patients with PD showed improved performance in activities of daily living after 14 sessions of balance training,<br>with no additional advantages associated with the Wii-based motor and cognitive training.
Fernandes et al. (2015)	RCT	EG = 8<br>CG = 7	EG: Motor-cognitive dual-task training<br>CG: Motor single-task training	12 sessions; 6 weeks; 60 min each	TUG,<br>UPDRS-III,<br>Pressure Platform,<br>RSCardsT,<br>TMT A and B	The results suggest superior outcomes for the dual-task training compared to the single-task training for static postural control, except in anteroposterior sway with eyes closed.

Lau et al. (2022)	RCT	EG = 9<br>CG = 9	EG: Training on a split belt treadmill combined with a first-person immersive video game targeting visuospatial skills and working memory.<br>CG: Waitlist control	12 sessions; 4 weeks; 30 min each	6 MWT,<br>TUG,<br>TUG Cognitive,<br>VF, SDMT	The use of immersive gaming technology to engage specific areas of cognition related to gait is feasible in PD. The treadmill training program paired with a customized interactive video game improved walking velocity in addition to non-significant but consistent improvements in other gait measures and cognitive performance in participants with early to mid-stage PD.
Lin et al., 2024	RCT	EG = 8<br>CG = 8	EG: Dual cognitive-walking Treadmill Training (DTT) – treadmill training with cognitive tasks displayed on a big screen in front of the treadmill<br>CG: Single Treadmill Training (STT)	25–45 min for each session, 2 times a week for 8 weeks.	UPDRS-III, FES, PGIC, PDQ-39, step length, walking speed, accuracy, reaction time	DTT can enhance cognitive function without compromising walking ability and also have real-world transferability.
Maidan et al. (2018)	RCT	EG = 30<br>CG = 34	G1: Treadmill training + Virtual Reality (targeting both motor and cognitive)<br>G2: Treadmill training	18 sessions; 6 weeks; 45 min each	Gait and prefrontal activations were assessed during 3 different walking tasks, MMSE, Go/noGo test, Stroop test, Catch game, UPDRS, f-NIRS	Prefrontal activation during usual and during more challenging walking conditions can be altered in response to 2 different types of training. Beneficial effects of treadmill training with virtual reality targeting both motor and cognitive functions on fall rates were found.
Wong et al., 2024	RCT	EG1 = 11<br>EG2 = 11<br>CG = 10	EG1: motor-cognitive training group<br>EG2: complex walking training group<br>CG: no additional training	40 min/session, 12-session over 6 weeks.	Prefrontal and premotor cortex activity, supplementary motor area activity, TUG, FES, MoCA, DST, VF, ROCF	The cognitive-motor training is suggested as an effective rehabilitation program to improve obstacle walking ability in individuals with PD.
Yang Y-R et al. (2019)	RCT	EG1 = 6<br>EG2 = 6<br>CG = 6	EG1: Cognitive dual-task gait training (CDTT)<br>EG2: Motor dual-task gait training (MDTT)<br>CG: General gait training/ control group	12 sessions; 4 weeks; 30 min each.	Speed cadence, stride length, double support time, stride time variability.	CDTT decreased double support time during cognitive dual task walking, and MDTT reduced gait variability during motor dual task walking in people with PD. In addition, the CDTT improved the speed, stride length, and double support time under motor dual task walking and single walking.

TUG: Up and go test; 10MWT: 10-meter walk test; DSB: Digit Span Backward, DSF: Digit Span Forward; VF: Verbal Fluency ; WAIS-III: Wechsler Adult Intelligence Scale; BAI: Beck Anxiety Inventory; WHOQOL_OLD: World Health Organization Quality of Life Measure OLD; MDS-UPDRS: Movement Disorder Society-Unified Parkinson's Disease Rating Scale Motor Examination; M-PPT: Modified Physical Performance Test; PD-CRS: Parkinson's Disease- Cognitive Rating Scale; TMT: Trial Making Test; VM: Verbal Memory; VIM: Visual Memory; EFs. Executive Functions; ToM: Theory of Mind; WHO-DAS-II: Worl Health Organization Disability Assessment Schedule II; RSCardsT: Rule Shift Cards Test; SDMT: Symbol Digit Modality Test; 6 MWT: 6 min walking test; MMSE: Mini Mental State Examination; f-NIRS: functional near-infrared spectroscopy; H&Y: modified Hoehn and Yahr Scale; GDS: Geriatric Depressive Scale; SVF: Semantic Verbal Fluency Test; RAVLT: Rey Auditory Verbal Learning Test; CDT: Clock Drawing Executive Test; PDQL: Parkinson disease Quality of Life Questionnaire; BBS: the Italian Berg Balance Scale; FIM: The Italian Functional Independence Measure; PDQ-39: the Italian 39-question Parkinson's Disease Questionnaire; UPST: The Unipedal Stance Test; MoCA: Montreal Cognitive Assessment; BESTest: Balance Evaluation Systems Test; FAB-it: Frontal Assessment Battery; FAS:F-A-S Verbal Phonemic Fluency Test; BDI: Beck Depression Inventory; STAI: State-Trait Anxiety Inventory; 2WMT: 2-minute walking test; ADL: Activities of Daily Living; TR: Telerehabilitation; FES: Falls Efficacy Scale; FSS: Fatigue Severity Scale; TUG: Timed Up and Go; SRT: Simple Reaction Time; FDS: Forward Digit Span; PGIC: Patient Global Impression of Change; DST: Digit Span Test; VF: Verbal Fluency; ROCF: Rey-Osterrieth Complex Figure

Table SII. Risk of bias assessment in quasi-experimental clinical trial design.

		**INTERNAL VALIDITY BIAS RELATED TO:**									
		**DOMAIN**	**Temporal precedence**	**Selection and allocation**	**Confounding factors**	**Administration of intervention/exposure**	**Assessment, detection, and measurement of the outcome**			**Participant retention**	**Statistical conclusion validity**
		**QUESTION N.**	**1**	**2**	**3**	**4**	**5**	**6**	**7**	**8**	**9**
**STUDY ID**	**OUTCOME**	**RESULT**									
Alves et al., 2018	functional mobility	Time 1	Y	Y	Y	Y	Y	Y	Y	Y	Y
		Time 2								Y	Y
		Time 3								Y	Y

Figure S1. Risk of bias assessment in randomised controlled trials

Study ID	D1	D2	D3	D4	D5	Overall
Chua et al. (2021)	!	+	+	+	+	!
El Semary et al. (2020)	!	!	+	-	!	-
Fernandes et al. (2018)	+	-	+	+	!	-
Lau et al. (2022)	+	+	+	+	!	!
Maidan et al. (2018)	!	+	+	+	!	!
Mariano Barboza et al. (2019)	+	+	+	+	!	!
Monticone et al. (2015)	+	+	+	+	!	!
Pompeu et al. (2012)	+	+	+	+	!	!
Terra et al. (2020)	+	+	-	-	!	-
Varalta et al. (2021)	+	+	+	+	!	!
Yang et al. (2019)	+	+	+	+	!	!
Das et al. (2024)	!	+	+	!	!	!
Del Pino et al. (2023)	-	+	+	!	!	-
Lin et al. (2024)	+	+	+	!	!	!
Wong et al. (2024)	+	+	+	+	!	!

+	Low risk
!	Some concerns
-	High risk

D1	Randomisation process
D2	Deviations from the intended interventions
D3	Missing outcome data
D4	Measurement of the outcome
D5	Selection of the reported result
